# Polymeric biomaterial-inspired cell surface modulation for the development of novel anticancer therapeutics

**DOI:** 10.1186/s40824-023-00404-8

**Published:** 2023-06-21

**Authors:** Ashok Kumar Jangid, Sungjun Kim, Kyobum Kim

**Affiliations:** Department of Chemical and Biochemical Engineering, College of Engineering, Dongguk University, Seoul, South Korea

**Keywords:** Polymeric biomaterials, Cancer recognition, NK cell surface modulation, Polymers for cell surface modification, Cancer immunotherapy

## Abstract

Immune cell-based therapies are a rapidly emerging class of new medicines that directly treat and prevent targeted cancer. However multiple biological barriers impede the activity of live immune cells, and therefore necessitate the use of surface-modified immune cells for cancer prevention. Synthetic and/or natural biomaterials represent the leading approach for immune cell surface modulation. Different types of biomaterials can be applied to cell surface membranes through hydrophobic insertion, layer-by-layer attachment, and covalent conjugations to acquire surface modification in mammalian cells. These biomaterials generate reciprocity to enable cell–cell interactions. In this review, we highlight the different biomaterials (lipidic and polymeric)-based advanced applications for cell–surface modulation, a few cell recognition moieties, and how their interplay in cell–cell interaction. We discuss the cancer-killing efficacy of NK cells, followed by their surface engineering for cancer treatment. Ultimately, this review connects biomaterials and biologically active NK cells that play key roles in cancer immunotherapy applications.

**Graphical Abstract:**

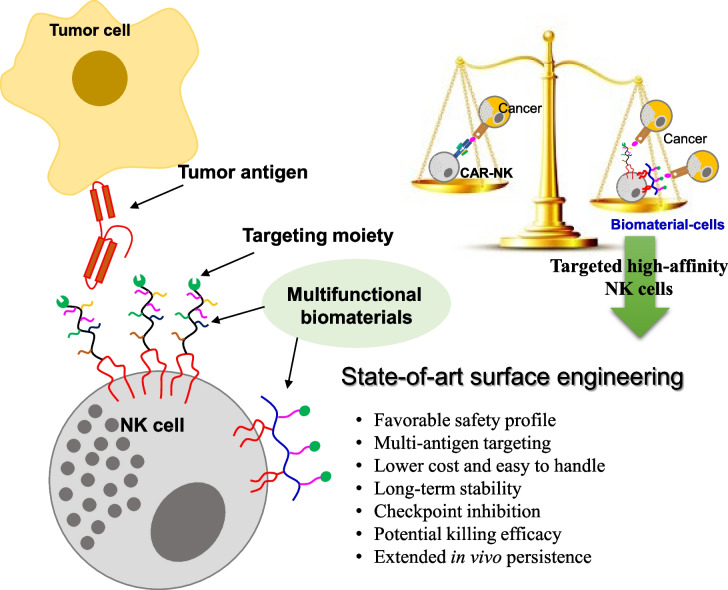

## Introduction

Mammalian cell membranes contain a sophisticated heterogeneous environment with various functional moieties of protein, carbohydrates, lipids, and other composites. These functional moieties participate in the identification of adjacent cells through physical contact-mediated direct cell–cell interactions and further dynamic signal transductions [1, 2]. Therefore, the presence of additional functional groups on cell surfaces could effectively manipulate cellular functions. Modification of cell membranes using suitable natural or synthetic biomaterials containing such functional moieties opens a new windows for selective cell–cell interactions [1].

**Fig. 1:**
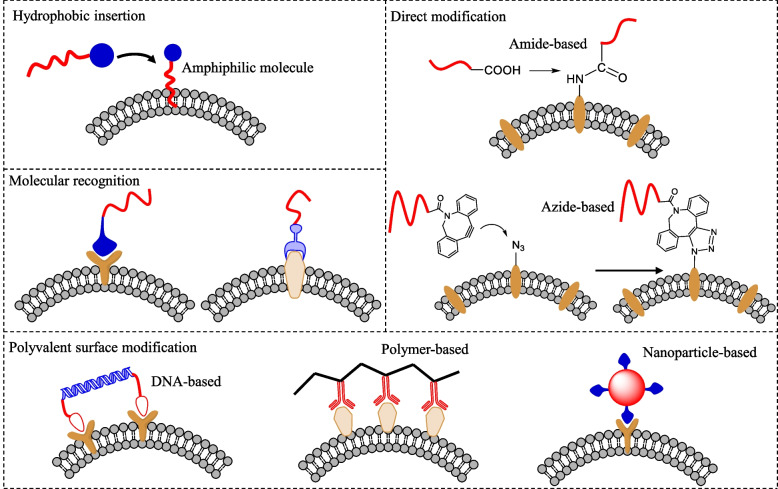
Scheme representing different approaches for cell surface engineering

In recent years, many cell surface engineering strategies have been investigated for the development of cutting-edge cell-based therapeutic agents (Fig. 1). These approaches have successfully modulated cell recognition, cell tracking, imaging, and cell-based immunotherapy [2, 3]. For cancer treatments in particular biomaterial-mediated surface modification techniques are undoubtedly clinically effective and robust. Among various tools for cellular surface modification, the anchoring of biomaterials into cell membranes inspires cell–cell and cell–extracellular interactions. Surface presentation of such specific functional moieties on cell membranes has been utilized to augment cellular intrinsic properties and improve surface-mediated cellular signaling.

Recently, the use of polymer-based materials for effective cell–coating has attracted considerable attention towards developing cell-based therapeutic products. However, the conventional cell–coating processes still present many technical limitations, including interferences in cell division, proliferation, and subsequent disruption in cellular structure [4, 5]. More importantly, undesired coverage of cell receptors/ligands and inhibited secretion of translated protein products by the addition of coating materials on the cell surfaces also downregulate intrinsic cellular functionalities.

Therefore, it is necessary to design and develop such biomaterials with significantly improved cellular surface properties that include: (1) the protection of cell architectures, (2) the preservation of signal releases such as cytokines and growth factors, (3) the stabilization of cell structure after coating, (4) enhanced cell–cell interactions, and (5) suitable degradation or dissociation of coating materials after coated-cell delivery, especially upon in vivo administration.

In this regard, anticancer treatments using surface-modified immune cells have been intensively investigated [6]. For example, chimeric antigen receptors (CAR)-modified T (CAR–T) cell is the most representative immune cell-based immunotherapy formulation for acute lymphoblastic leukemia treatment [7, 8]. Further efforts have focused on improving the anticancer efficacy of CAR–based strategies via increased target specificity and subsequently reduced associated side effects. Facilitated tumor recognition and antigen-specific targeting could extend the therapeutic benefits of CAR-based cellular therapeutics [9]. This is often accomplished by expressing bi-specific/tri-specific recognition moieties that target different antigens in tandem, and bind with single signaling endodomain. Subsequently, a similar surface engineering technique using precise genetic modification has been integrated into NK cell-mediated anticancer immunotherapies as well [10, 11]. NK cells exhibit potential toxicity against various types of solid tumors as self-therapy agents, due to the absence of MHC class I molecules on their surfaces [12, 13]. NK cell can potentially kill the cancer cells through two different methods: (1) direct cancer cell killing through the formation of immunogenic synapse between host and guest cells, and (2) indirect cancer cell cytotoxicity through activating secretory lysozyme containing perforin and granzyme. In addition, many NK cell receptors are also involved in inducing cytotoxicity in cancer cells through selective cancer cell recognition [14]. Cell cytotoxicity predominantly involved through Fas ligand (FasL) and tumor necrosis related apoptosis induced ligand (TRAIL), which presents on both host and guest cell surface, can trigger intracellular caspase activation [15, 16]. The activated NK cells could potentially kill the cancer cells through perforin and granzyme secretion [17–19].

**Fig. 2:**
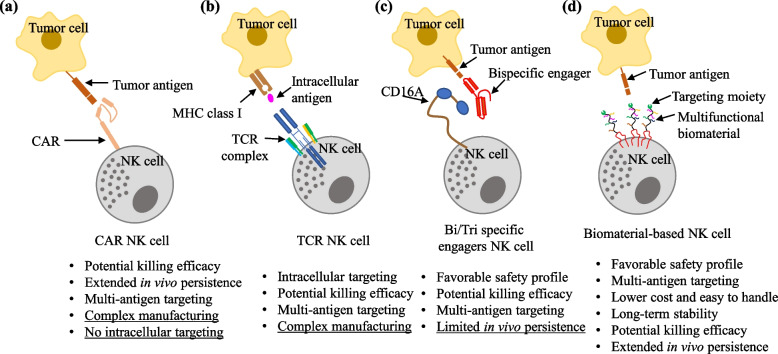
Different strategies enhancing NK cell activity to redirect tumor cell killing efficacy

Recently efforts have been made to enhance NK cell fitness and antitumor functions, to express an extracellular receptor and functional CD3/TCR signaling. Such studies have demonstrated that, these cells can target different tumors with potential efficiency, specificity and safety profile (Figs. 2a-c). However, these strategies still have some limitations, like complex manufacturing, HLA-restricted killing, and in vivo persistence [20–22].

Motivated by the potential efficacies, encouraging results and their limitations, there is growing interest in the design of surface-engineered NK cells using functional biomaterials for potential cancer immunotherapy (Fig. 2d). Therefore, surface-coated NK cells via multifunctional biomaterials with proximal recognition moieties have become one of the most fascinating research areas. As per the previous reports, the use of non-genetic modulation using these biomaterials could be a safer and easier way to manipulate the composition of cell membranes [23]. Therefore, the incorporation of multifunctional biomaterials onto NK cell surfaces augments tumor–targeting ability and regulates subsequent immune responses through cell-contact biophysical phenomena [24].

Recently, several substantial studies have reported the efficacy of biomaterials on various therapeutic treatments; however, these molecules have hardly been recognized as strong target agents or medications [25]. In a few studies, researchers also found that the multifunctional biomaterials can bind with protein/lipids/small molecules, and make use of these molecules in cell–cell recognition, immune response, and cell signaling. Therefore, a careful selection of biomaterials for cell surface modification, which might be used as a possible therapeutic system, is necessary for efficient NK cell surface engineering for cancer therapy. In this review, we aim to provide an overview of (1) the utilization of prominent cancer recognition moieties, (2) practical synthesis strategies of cell membrane anchors, and (3) the latest advances in the design of multifunctional biomaterials for ex vivo NK cell surface modification.

## Cancer recognition

In-depth understanding of the composition and related interaction of cell surface components is necessary to improve target-specific binding between immune cells and tumor cells. By augmenting a variety of synapse interactions, accurate regulation in site-specific binding could be achieved without intervention [26]. To this end, applying additional recognition moieties into immune cells could eradicate rapid transformation of the malignant cell population without affecting normal cells [27]. Specific use of cancer recognition moieties monitored real time therapeutic efficacies of modulated immune cells with personalized preventions. In past years, various recognition moieties, including small molecules, proteins, peptides, aptamer, glycans, and vitamins, have been effectively used to identify and target cancer cells [28].

Recognition moieties could be conjugated with a series of biomaterials for preferential integration into solid tumors. Such conjugated recognition moieties afford potential advantages over conventional vectors, including selective attachment, the avoidance of biological barriers, and enhanced permeation and retention (EPR) effect [29]. Several cancer targeting moieties have been used to facilitate cancer recognition [30–32]. In this section, the most potent acid molecule-based cancer recognition moieties, i.e., folic acid, lactobionic acid and boronic acid, are discussed.

### Folic acid recognition by folate receptors

**Fig. 3:**
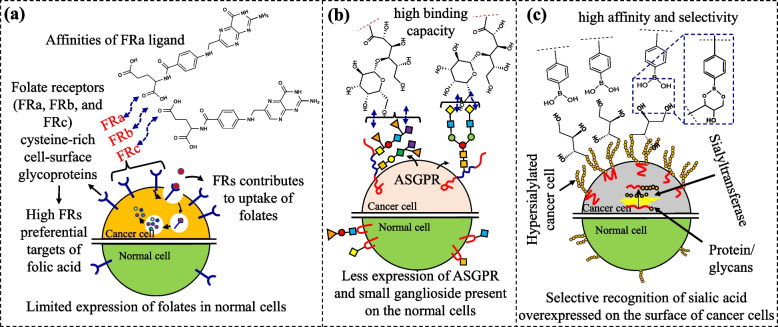
Scheme representing the role of (**a**) folic acid, (**b**) glycan lactobionic acid, and (**c**) phenyl boronic acid as a cancer recognition moiety for selective anticancer targets

Folic acid (FA) is a vitamin B9-based biomolecule and a multicomponent moiety that contains glutamic acid, teropterin, and *para*-amino benzoic acid. Folic acid (FA), a vitamin B9-based biomolecule, has been most widely used for different cancer targeting, based on its selective binding affinity to folate receptors (FRs) overexpressed in many cancer cells (Fig. 3a). For example, the overexpression of FR–α is generally observed in over 40% of cancer cells, while FR–β is overexpressed in macrophages [33]. Hence, these overexpressed cysteine-rich glycoprotein-based receptors showed high affinity with surrounding the FA and the binding of FA to FRs promotes the clustering of ligand–receptor complex in cellular surfaces, resulting in internalization via endocytosis [34–37]. At endosomal pH, FA release from receptors and subsequent transport by proton-coupled folate transporters in the cytoplasm occur. Folates also regulate cellular migration, and are associated with tumor progression. It has been reported that the introduction of antifolates in ovarian cancer cells has significantly reduced cell division, independent growth, and the adhesive properties of cancer tissues [35, 38, 39]. Furthermore, FA increases cell proliferation and apoptosis against cancer cells overexpressing FR through JAK–STAT signaling pathway [40].

Thus, FA would be considered an interesting cancer cell recognition and targeting ligand, despite its selectivity based on less expression level in normal cells and more efficient internalization to target cancer cells.

Alternatively, designing a FA-based platform to FRs targeting could be a better approach to treat different cancer cells. FA has many advantages over the other targeting moieties: it can easily bind with small molecules present at the cancer cell membrane; it can easily internalize; and it is less overexpressed in normal tissues. The advantages of FA make it a good targeting ligand that selectively binds with cancer cells [41, 42].

However, the FA moiety has been widely used for cancer cell recognition where it also plays a vital role in cell–cell adhesion. Previously, FA was used for cell surface modification to induce cell–cell interaction through FRs at the cancer cell targeted site [35, 43]. In terms of advantages, the FA moiety would be applied for cell surface modification that could enhance the cell–cell interaction. Application of FA conjugated to immunoglobulin (IgG) applied activates NK cells, and selectively binds to FR overexpressed cancer cells [44]. The FA–hepten conjugate is applied to the immune cell to selectively kill cancer cells. It is also reported that the therapeutic efficacy is bimodally dependent on the amount of FA–hepten and showed potential toxicity through the bridge between the cell surface FA and receptors overexpressed in cancer cells [45]. The FA-modified cells would rapidly interact with cancer cells [46]. From another prospective, FA has a few disadvantages, such as a high amount increasing the toxicity toward normal cells and those cells that use them for surface engineering. The high dose of FA also reduced the cytotoxicity of NK cells in mice [47]. Therefore, it is essential to precisely select and design a biomaterial system that can be utilized for NK cell surface modification for cancer immunotherapy.

### Glycan lactobionic acid recognition by glycans receptors

Cancer cells have different glycan coatings than normal cells because their glycoproteins and glycolipids are fundamentally altered in cancer cells. Glycan structures are formed by the coordinated action of several enzymes that can catalyze the addition or removal of glycans that are covalently bound to proteins or lipids [48]. The glycans associated with cancer cells, like Lewis’s antigens, sialylated structures, and glycol proteins, are frequently part of the membrane–secreted tumor proteins. For example, asialoglycoprotein receptors (ASGPR) are primarily overexpressed on a variety of tumorous cells, including hepatocellular carcinoma, colon, breast, lung, and prostate cancer cells [49]. These receptors selectively bind with galactosyl residues of the surrounding molecules, and help further the endocytosis process (Fig. 3b). The unique interaction between these galactosyl moieties and cancer cell receptors allows them to be potential cancer targeting ligands [50].

In another way, cancer cells also display the abnormal expression of Galectins (a lectin-based protein) that specifically binds, in addition to aberrant glycan expression. Galectins may be emitted by a wide range of cancer cells, and potentially impaired the function of immune cells like the effector function of T cells, suppressed myeloid cells, and modulated NK cells activity through binding to specific glycans overexpressed on the immune cells [51]. The cancer cell aggression and metastatic correlated with Galectins expression and blockage in tumor microenvironment enhanced the effective function of CD4 + and CD8 + T cells [52]. Additionally, Galectins expression also inhibits the MICA ligand of the NK cell and glycol-dependent interaction [53]. Because NK cell ligands also contain the glycan structure, the binding between NK cell glycan structure and Galectins leads to NK cell evasion [54]. Therefore, the down regulation of Galectins or specific targeting of glycan receptors in solid tumors can lead to tumor suppression and growth inhibition. In another aspect, a strategy that was able to prevent the interaction between inhibitory immune receptors and glycans could serve as better anticancer therapy. The metabolic competition between immune and cancer cell glycans hindered immune cell functions through defective IFN–γ production [55].

Glycol monomer structures such as gluconic acid and lactobionic acid (LBA) act as a Galectin-inhibitors as well as offiring specific binding abilities [56, 57]. Among the monomers, LBA is a sugar molecule also shows the potential inhibitory effects against Galectins, and can also improve tumor immunity in cancer cells [58]. In past years, LBA-based drug delivery systems were specifically utilized for hepatocellular targeting [59–61]. It is well known that lectin receptors can easily recognize and endocytose glycol-based drug carriers or sugar-based ligands, especially tumor cells with overexpressed Siglecs receptors through the high binding affinity toward sugar moieties [62].

In the case of NK cells, sugar-mediated modification improved the selective binding ability of NK cells to target cancer cells [63]. Indeed, therapeutic modification of glycol, such as blockage of overexpressed glycans through metabolic memetics, can suppress tumor growth. The tumor suppression can be enhanced presumably due to NK cell activation resulting in decreased Siglecs triggering [48]. In another direction, glycans have also been used for NK cell activation through direct protein glycosylation [64, 65] or biomaterials-mediated glycosylation [66]. However, direct glycosylation onto NK cell surfaces requires the precise identification and selection of well-defined glycans, glycoproteins, and glycopeptides. Therefore, glycol-based biomaterials for NK cell surface engineering has become a rational approach that utilizes glyco-receptor mediated endocytosis [67–70].

The cell surface with overexpressed sugar moieties could be utilized to modulate cell–cell interaction, transduce signals, and regulate immune response. As compared to normal tissues, aberrant glycosylation overexpression in cancer tissues could be a potential biomarker for cancer cell recognition. Therefore, utilizing LBA as a glycosylation binding agent could be a better therapeutic approach that directly promotes cancer cell suppression.

Glycosylation also affects the immune response of cancer cells. Immune cells are mainly regulated by immunoglobulins and lectins receptors on their surface membranes. Glycosylation of immune cells and cancer cell receptors plays an important role in cancer immunotherapy. For example, in several different forms of cancer, proper ligand–receptor interaction and subsequent activity in the antitumor immunity depend on the glycosylation of PD–1, PD–L1, or B7–H4 [71, 72]. Cancer cells with a high expression of glycoproteins are also susceptible to immune cell cytotoxicity. For example, a high expression of sialyl-based fructosyltransferase 3 (a glycoprotein) onto HepG2 cells resulted in elevated binding affinity with lectin-like receptors of NK cells, and achived downstream NK cell–mediated cytotoxicity [73].

Cancer cells included the expression of O-glycans, N-glycans, sialylated and various fucosylated-based glycans on the cellular surface. Hence, cancer cell overexpressed glycans play a key role in cancer therapy including cancer target, diagnosis and treatment [74]. It is also reported that, the cancer cells expressed glycans facilitate the interaction between cell surface and its surrounding tumor microenvironment through the development of cancer biomarkers like proliferative signaling, resistance to cell death, immune evasion, and angiogenesis. Moreover, cell surface glycoproteins also protect the cell membrane from physical stress and the chemical aspects of cell surface dynamics [75]. Therefore, because of their unique features and their wide range of applications in cancer, glycans have become a promising and fascinating research area in the context of tumor-targeted therapy.

Carbohydrate-based molecules can play important roles in cell–cell interactions. These molecules can self-arrange or be localized near recognized target cells, where they act as a binder with the cancer cell. Saccharides will bind with specific complimentary glycans, or with lectins of target cell. Owing to the specific structure of these molecules, they can modify the immune signals and stability of PD–1.

Glycans such as mannose, galactose and lactobionic acid (LBA) can be used as targeting ligand for hepatocytes, endothelials, and macrophages. Among these carbohydrates, LBA disaccharide molecule, shows good binding affinity with hepatocytes overexpressed ASGPRs receptors through LBA–ASGPR complex formation [59, 76]. The overexpressed ASGPRs promote clathrin-mediated endocytosis during the interaction. ASGPRs mainly consist of two different polypeptide subunits which contain carboxylic groups (–COOH) at the terminal position. Therefore, the hydroxyl (–OH) group of LBA and –COOH of receptors enables the interaction followed by complex formation. Some carbohydrates molecules exhibit cellular and immune response via the enhanced production of proinflammatory modulators [77]. Like other carbohydrates molecules, LBA is also capable of stimulating the immune synapse. Hence, LBA, an effective epitope, could be a promising targeting sugar molecule for effective cancer cell recognition, and treatment via contact with glycoprotein binding.

### Boronic acid recognizes sialic acid and carbohydrates on cell surface

Sialic acids (SAs) belong to the sugar family (N–Acetylneuraminic acid) with a nine-carbon backbone structure [78]. The SAs are typically present on several cellular surfaces and secreted glycan molecules on cancer cell surfaces [79]. The aberrant overexpression of SAs on cancer cells can lead to new interplay with immune cells, resulting in the blocking of immune cell activation. The thick coating of SAs on the surface of cancer cells prevents the immune system from eliminating tumors. Siglecs are the sialic acid-binding immunoglobulin-like lectins that can specifically bind to the SAs. Siglecs can be further divided into two sub-categories based on sequence similarity and evolutionary conservation [80]. The first Siglecs group consists of sialoadhesin (Siglec–1), CD22 (Siglec–2), MAG (Siglec–4), and Siglec–15, while the second group consists of CD33 (Siglec–3), Siglec–5, Siglec–6, Siglec–7, Siglec–8, Siglec–9, Siglec–10, Siglec–11, Siglec–14, and Siglec–16 [81]. Most Siglecs serve as activating receptors and are involved in cell–cell interactions, while the majority of Siglecs are responsible for mediating inhibitory signals [82]. Siglecs can effectively bind with target cells via two different interactions: (1) trans-interaction and (2) cis-interaction. The trans-interaction improved binding between effective and target cells, whereas the cis-interaction acted as a masking function on the cell surface, significantly inhibiting SAs binding efficacy. In another direction, abnormal overexpression of SAs on cancer cells could promote cancer growth and proliferation [83], manipulate extracellular matrix interactions, cell–cell interactions, and cell-membrane decoration [84], and effectively overwhelms towards immune cell response and encourage cancer metastasis [85]. Thus, it may also play a role in the molecular mimicry that allows cancer to avoid host cell immune responses.

The hypersialylation (overexpressed SAs) on the cancer cell makes major ligands for the Siglecs binding (present on the immune cell surface). Siglecs and SAs bind to support immunosuppressive signaling, and provide protection to tumor cell. For example, Siglecs–7 and Siglecs–9 present on the NK cell surface bind with SAs on the cancer cell, inhibiting the NK cell toxicity towards cancer cells [86–88]. In the tumor environment, sialyl–Tn (sTn) antigens, sialyl Lewis x (sLex) antigen, GD2 disialoganglioside with the sialyl Lewis A epitope (sLea) and sTn epitope, have been used for cancer biomarkers and treatment [89]. Hence, the cancer cell immune response is majorly affected by the Siglecs [90–92]. For example, Hudak et al. [86] reported that Siglec–7, an immunoglobulin-like lectin 7 excessively overexpressed in cancer cells, inhibits the cytotoxicity of NK cells by bonding with sialic acid. The immunoreceptor tyrosine-based inhibitory motif (ITIM) found in both Siglecs–7 and Siglecs–9 attracts SHP phosphatases to the region of activation, and prevents the kinase phosphorylation cascade from proceeding. In this study, they also reported that surface–engineered NK cells with increasing ligand density coreceptors would be sufficient to suppress cell killing. Here, a modified synthetic glycopolymer was introduced on the cancer cell surface, and shows that increasing sialylation on cancer cells inhibits the NK cell activation through Siglecs–7 binding. In another sence, Nicoll et al. [93] demonstrated that, Siglec–7 specifically interacts with CD–33 related sialylated probe of cancer cells, and inhibits NK cell cytotoxicity. Here, transfection-based methodology was used to examine the impact of siglec–7–gangliosides GD3 interactions on NK cell killing activity. Siglec-7 is normally concealed on NK cells, but sialidase can make it visible so that it can interact with GD3 on target cells to stop killing. In another way, the expression of GD3 on the cancer cells also increased the siglec–7 mediated killing efficacy.

In recent years, phenylboronic acid (PBA)-based biomaterials have been used to recognize characteristic carbohydrates present on cancer cell surfaces [94–100], due to the selective binding of PBA with various sialic acids in the tumor microenvironment (Fig. 3c) [101–106]. Since this binding ability of boronic acid under different pH conditions was first reported in 1959 [107], a new design strategy has been suggested to develop PBA-decorated biomaterials for cancer treatment [108, 109]. Superior in vitro and in vivo antitumor activities in restricting tumor growth and increasing the survival time of tumor–bearing mice were obtained via PBA–SAs mediated interaction[102, 106, 110]. These studies suggested the potential targetability and pH-triggered drug release profile from PBA–SAs biomaterial. Owing to the ability to form 1,2 and 1,3 diols boronic ester, PBA can also be used in saccharide biosensors applications. Similarly, PBA and SAs can form the reversable borates, which promotes the uptake of cancer cells [111]. Elsewhere, after endocytosis in the tumor cell, the dissociation of the boronic ester complex leads to a decrease in pH level, and an increase in ATP level [105, 112, 113].

Therefore, by introducing PBA on immune cells, it can selectively bind with overexpressed sialyls or siglecs moieties, and can increase the immune cell cytotoxicity toward targeted cells. In other words, PBA would be an effective moiety, and its materials can also be anchor to NK cell membrane; thus, it would also be a promising targeting ligand for selectively targeting cancer cell binding and the treatment of tumor cells.

## Synthesis strategies of cell membrane anchors

Today, the ex vivo surface engineering of NK cells by means of modified biomaterials has become a powerful technique for tailoring cell surface propertie, and thereby augmenting the intercellular interactions between NK cells and surrounding target cells. This biomaterial-mediated cell membrane modification could be regulated by the structure of biomaterials, density, and functional groups of biomaterials on NK cell surfaces. Therefore, the design of unique biomaterials without affecting intrinsic cellular properties has become a challenging task. Primary prerequisites for the development of these biomaterials for ex vivo cell surface engineering is the selection of proper anchoring moieties to facilitate membrane binding and presentation onto NK cell membranes. Subsequently, the fabrication of such biomaterials could be initiated by a series of conjugation chemistries including click chemistry, coupling reaction, michael addition, and azide coupling reaction.

Therefore, this section summarizes current investigations using lipid–PEG molecules and polymer conjugates for cell surface modification. In addition, new engineering strategies for designing functional biomaterials specifically for NK cells are discussed.

### Lipid–PEG conjugates act as direct anchors for cell membranes vector

**Fig. 4:**
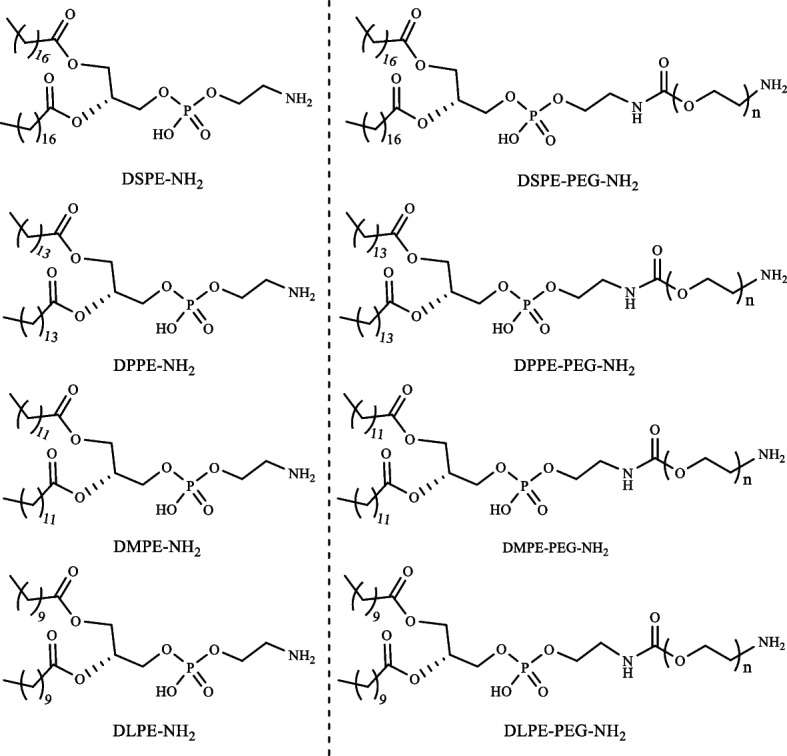
Commercially available lipids and their PEG-based derivatives can be utilized to develop cell–coating biomaterials

In recent years, lipid-PEG derivatives were successfully used for mammalian cell membrane surface modifications [114–116]. Lipids can be easily anchored on the cell membrane by hydrophobic interactions with the lipid bilayers of target cells. However, the selection of lipid chain should be preciously determined. As the dynamic and heterogeneous nature of cellular membrane components (i.e., a variety of biological molecules, lipids, and proteins along with intrinsic negative charge and hydrophobic cavity in cell surfaces) can be affected by the lipid insertion [117, 118]. Therefore, charge-based electrostatic interaction and lipid-mediated hydrophobic insertion have been used as major driving forces for the insertion and presentation of biomaterials onto target cellular surfaces. More importantly, the incorporation of such lipid–PEG conjugates on the cell membrane was greatly affected by the length of lipid chains or subsequently modulated hydrophobicity [119, 120]. The incorporation of lipid–PEG on the cell membrane is mainly affected by the length or hydrophobicity of conjugated lipids. Figure 4 describes representative commercially available lipids and PEG derivatives that could be utilized for the design of various cell–coating biomaterials. The anchoring of lipid–based oligonucleotides is a potential tool for cell engineering, and has a wide range of biomedical applications. The specific and unique amphiphilic structure of lipid-based oligonucleotides enables them to self-assemble and potentially monitor cell behavior [121]. Jin et al. synthesized DNA-based phosphorylated lipid (DNA–lipid–P) conjugates for cell membrane anchoring varying lengths of carbon chains (i.e., C6, C9, C12 and C15) in lipid moieties. The anchoring efficiency of synthesized phospholipids onto HepG2 and U–2 OS cells increased when C15 was conjugated. In addition, phosphorylation of this lipid conjugates also influenced the membrane anchoring as compared with non-phosphorylated lipids through alkaline phosphate (ALP)–dependent cell membrane anchoring. With less hydrophobicity, DNA–lipid–P exhibits relatively minimal interactions with the cell membrane in the absence of ALP. Dephosphorylation-induced with increasing hydrophobicity, DNA–lipid–P adheres to cell membranes that were highly ALP-expressing [122].

The development of modified lipid–PEG anchors is an easier and more rapid method for various cell membrane anchoring. The terminal moiety of PEG can be further modified to make a biocompatible surface anchor. Kato et al. developed biocompatible oleyl (single and double– tailed) chain-based PEG conjugates for various cells, i.e., NIH3T3, 32D, Ba/F3, hybridoma 9E10 cell–coating [123]. The two–tailed lipid anchor exhibited superior membrane anchoring ability, as compared to the single–tail lipid, implying that increasing the hydrophobicity of the anchoring materials improved the interaction with cell membranes, and resulted in rapid anchoring ability with high retention time. However, a few cells did not express on the cell membrane, so gene transfer methods show some limitations. Therefore, to design protein–enriched surface–based cells, anchoring lipid materials were further conjugated with some protein, i.e., streptavidin, antibody, and EGFP. The proteins-based anchoring lipid conjugates were successfully coated on the cells, and did not show any sign of toxicity.

The soluble part of the polymer on the living cell surface has been used in various biomedical applications. Cationic polymers (poly–lysine and polyethylene amine polymers) are used as gene delivery applications. However, due to the presence of the anionic charge on the cell membrane, the cationic polymer interacts strongly and shows cytotoxicity to living cells [124–126]. Therefore, anionic (poly vinyl alcohol) or neutral polymers (polyethylene glycol) can be applied to the cell membrane to induce cell–cell interaction or cell fusion. Owing to the absence of an anchoring moiety in both polymers, after conjugating a lipid/hydrophobic moiety, these modified polymers are expected to anchor the cell membrane. Lipid-conjugated synthetic polymeric derivatives exhibited a similar anchoring capability onto cell membranes. The lipid–conjugated synthetic polymeric derivatives can be applied to cell membrane by three different methods: (1) Lipid–polymer polymers can directly anchor to the cell membrane through hydrophobic insertion, whereas lipid moiety anchors to cell membrane and polymeric moiety to outside the membrane. (2) PEG conjugated with functional moiety (i.e., NHS) can be used for direct covalent modification of the cell membrane. (3) Ionic polymers (cationic or anionic) can be used through electrostatic interaction on the cell membrane, whereas ionic polymer can be attached with cell membrane functional moieties [127–129].

The interaction between the cell membrane surface and the polymer has major influence on the uptake and exclusion. The different modes of interaction also affect the dynamics and stability of modified polymers on the cell membrane. To improve the acceptance rate of therapeutic cells, the modified synthetic polymers are used to enclose cell surface antigens and immobilize bioactive molecules on the cellular surfaces [130]. Modified synthetic polymers i.e., hydrophobic containing (PEG–lipid, PVA–alkyl), charged polymers (cationic PEI and anionic PVA), and NHS-modified PEG were taken to modified living cell membrane. Each modified polymer acted differently with changing the surface availability of ligands. The PEG–lipid and PVA–alkyl anchored through hydrophobic interaction, charged polymers anchored through electrostatic interaction and NHS–PEG formed the covalent bonds with available surface membrane proteins. After successfully applying all the polymers to CCRF–CEM or HEK293 cell membrane, the PEG–lipid, PVA–alkyl, and NHS–PEG polymers show rapid homogenous surface coating, while PEI polymer destroyed the cell membrane integrity. Cationic PEI polymer strongly interact with cell membrane and shows toxic effects to cells [131, 132].

**Fig. 5:**
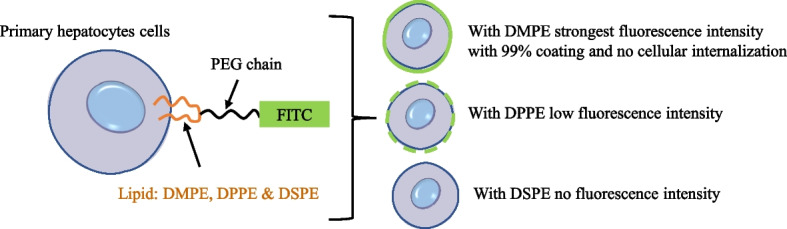
Hydrophobicity inducing surface coated primary hepatocytes cells. The primary hepatocytes cells coated with different FITC–labbled Lipid–PEG conjugates

The lipid composition and chain length have been considered important for regulating cell surface anchoring [133]. Figure 5 demonstrates a series of lipid–PEG conjugates with different carbon chain length, including PEG–DMPE (C14), PEG–DPPE (C16), and PEG–DSPE (C18). After incubation of hepatocytes cells with different lipid-based conjugates, PEG–DMPE (C14) exhibited uniform presentation with the highest anchoring efficiency on hepatocyte cell membranes. As compared to DMPE, the DSPE and DPPE–based conjugates were not sufficiently present on the cell membrane [134]. However, the effect of carbon chain length in lipids on cell surface anchoring should be precisely controlled, since controversial results were also reported. When two tail lipids conjugated with single–strand DNA oligonucleotides were applied for Jurkat cell surface anchoring, C16 lipid–DNA exhibited faster anchoring, compared with C18 lipid–DNA conjugates [135]. Similarly, Lee et al. have designed an antibody drug conjugate (ADC), i.e., trastuzumab emtansine with amine terminated DMPE–PEG to modified NK cell surface membrane. The designed ADC was successfully embedded on NK cells through hydrophobic interaction between the lipid–bilayer and anchoring lipid moiety [136].

The limited chemical space and retention of lipid anchors on the cell membranes is the structural parameter of different anchors. The passive exogenous insertion of different lipid motifs or chemically defined structures i.e., single–chain lipid, double–chain lipid, cholesterol, and vitamin E through hydrophobic insertion could be an alternative approach to modify the cell membranes [119]. The different lipidic moieties show that different–different anchoring efficacy depends on the hydrophobic nature of the anchoring molecules and cell membrane [137]. To check the cell–coating efficacy of different lipids, Uvyn et al. synthesized dinitrophenol (antibody recruiting motifs) functionalized with single–tail lipid, two–tail lipid, cholesterol, and PEG–biotin as cell membrane anchoring moieties. After incubating the different lipid–conjugates with CT26 cancer cells for 2 h, the two–tail lipid–polymer showed homogeneous coating on the cell membrane as compared to other conjugated motifs [138].

The incorporation of additional biomolecules (such as peptide or surface proteins) could augment cell–cell adhesion of surface–coated cells. One example is benzylguanine (BG) functionalized DSPE–PEG tagged with SNAP–tag protein for the cell membrane coating. The cell surface bounded proteins are mainly critical for the cell signaling, cell–cell communication and cell recognition properties. Therefore, to simplify this issue, Rudd et al. used benzylguanine (BG) functionalized DSPE–PEG tagged with SNAP–tag protein for the cell membrane coating. This method simplifies and controls the phospholipid protein expression on cell membrane, and would be a promising method for proteins targeting cellular membranes [139].

Similarly, peptide-conjugated lipids could also be applied to facilitate cell–cell attachment. Here, by introducing PEG–lipid on the cell membrane coating, the cell–cell attachment was significantly increased through peptides interactions. Two different type of oligopeptides EIAALEK (fuE3), and KIAALKE (fuK3) sequences, were conjugated with the end group of lipid-PEG moiety and used specifically for heterodimeric interaction between cells [140]. Interestingly, photocleavable materials in lipid–PEG conjugates offer light responsive cell–adhesion ligands [141–144]. It has also been reported that the cell membrane modified with PEG single–chain lipids could show better stably anchoring profile, as compared to dual–chain lipid–based PEG conjugates [145]. Based on such versatility, light induced PEG–lipids serve as turn-off-type cell-anchoring substances for targeted cell attachment to locations that are not exposed to light [146].

The non-invasive remote regulation of cell anchoring to biomaterial has been made possible by the development of stimuli-responsive cleavable materials that are sensitive to heat, voltage, and light. The photo-sensitive materials are the most promising, and offer the ability to control cell surface modification even at single cell level accuracy. Therefore, Yamahira et al. conjugated a photocleavable molecule (4-[4-(1-hydroxy-ethyl)-2-methoxy-5-nitrophenoxy] butyric acid) with PEG–single chain lipid, which was further conjugated with additional lipid moiety to make a dual–lipid based PEG conjugate. Here, for the anchoring comparison, different chain length lipids were conjugated with PEG–single lipid conjugate. The insertion of second lipid chain to PEG–single lipid prevents cell membrane anchoring. Before exposer of light, dual chain lipid–PEG inhibits the cell membrane anchoring and after the exposure to 365 nm light, cell anchoring switched off on the same surface [147].

### Polymer-based conjugates anchor cell membrane

Cell membrane anchoring using a variety of natural or synthetic polymers provides new opportunities for bioengineering applications. Polymer derivatives with multifunctional moieties could also be easily introduced on the cell membrane with the aid of bioactive membrane anchors [148, 149]. In recent years, three major strategies have been suggested for cell membrane anchoring: (1) amphiphilic polymers and PEGs via hydrophobic insertion, (2) covalent conjugation of polymers with active functional components in cell membrane surface, and (3) layer-by-layer surface deposition by ionic polymers.

However, the covalent conjugation and layer-by-layer cell surface methods have limitations such as disturbing the signaling functions of surface proteins and compromising efficacies. The covalent surface modification has been achieved through only chemical, enzymatic, or metabolic treatment which introduce a variety of functional groups including azide, amine, carboxylic, biotin, N-hydroxy succinimide ester, maleimide, and ketones. However, these chemical methods would perturb the cell membrane or cells, and can also disturb physiological signaling processes. Similarly, the layer-by-layer deposition of ionic polymers has exhibited detrimental effects toward target cells due to the cytotoxicity of such ionic materials and random surface coverage inhibiting signal binding or secretion through membrane surfaces.

**Fig. 6:**
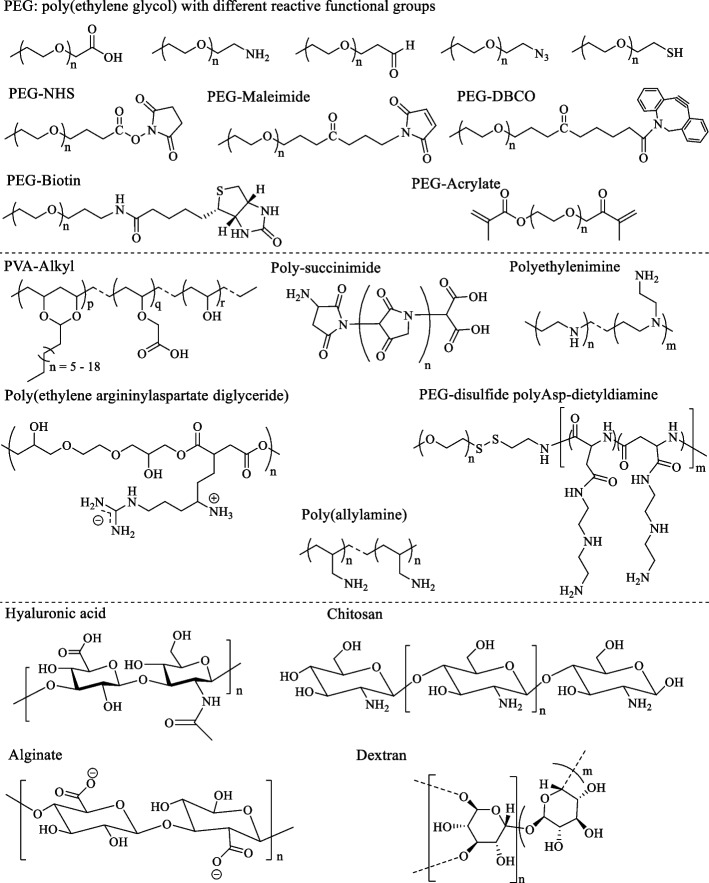
Chemical structures of different carboxylate, amine, aldehyde, azide, thiol, maleimide, DBCO, and alkene-based biopolymers that can be utilized for cell membrane modification

Synthetic polymers (PEG, PEI, PAA and PVA) [150] and natural polymers (alginate, chitosan, hyaluronic acid and dextran etc.) [151–153] are good biopolymers with biodegradable, biocompatible and well-arranged structural properties. These polymers could be utilized for the surface modification of NK cells for biomedical applications (Fig. 6). However, the lack of ideal cell anchoring and cell mimicking functional moieties render the surface features of these polymers inappropriate for cell surface engineering.

The negative charge on the cell membrane helps for electrostatic binding sites for the charged polymers. The negative charged based-polymers cannot attach to cell membrane due to charge repulsion forces while the cation-based polymers can bind with cell membrane due to opposite charge binding. Different studies based on polycations were used to modify mammalian cells, like poly–L–lysine, PEI and PAH [154–156], since the interaction between cationic polymers and cell membrane shows potential cytotoxicity [157]. Additionally, incubating cells with polycationic polymers forms a speckled cell covering by electrostatic adherence to the cell surfaces. Therefore, the suitable modification of these polymers could be utilized as cell–coating biomaterials.

**Fig. 7:**
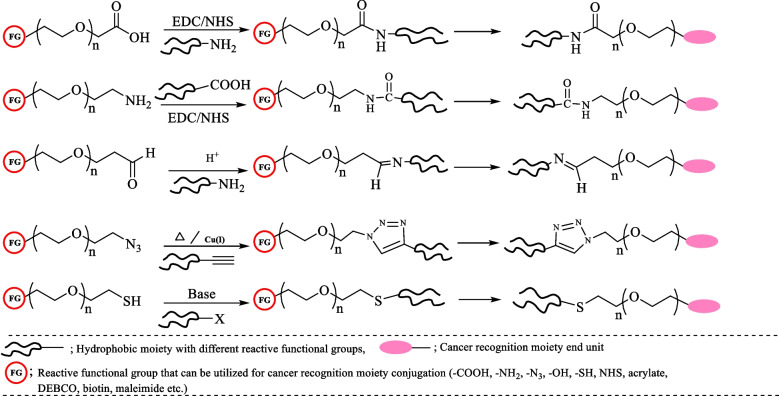
Schemes representing the general synthesis strategies of different hydrophobic-PEG biomaterials for cell–coating applications

Hydrophobic modification of polymers provides an opportunity to change the properties of these biopolymers to achieve a regular cell–coating without altering the cell membrane physiology [5, 158, 159]. The availability of versatile polymer modification methods such as EDC/NHS-based coupling reactions, NHS–ester based coupling, or Michael addition, can be used for biopolymer modification. As discussed in Sect. 3.1, the different types of lipids, including tail basis and C-chain length basis lipids, can be utilized to design coating platforms. However, two tail lipids, i.e., DSPE– and DMPE–based polymers, showed homogenous cell coating efficiency [138]. Therefore, conjugation of DSPE and DMPE lipids with polymers could provide better cell–coating efficacy, and enhance cell–cell interaction (Fig. 7).

Systematic surface engineering without interfering with NK cells’ own death receptor ligands is crucial for effectively augmenting cell recognition ability and the therapeutic efficacy of NK cells [160]. However, previously reported cell–coating materials still exhibit various limitations to achieving sufficient therapeutic efficacy of surface-coated NK cells. Due to the rapid dynamic nature of NK cell membrane, a single usage of cholesterol or one-tailed lipid molecules could be internalized into NK cell cytoplasm, and induce insufficient membrane-binding capability.

To this end, new design strategies for multifunctional polymeric anchors for enhanced NK cell surface engineering should be suggested: (1) two-tailed lipid molecules for efficient cell anchoring efficacy, (2) poly(ethylene glycol) (PEG) for increased solubility of the fabricated polymeric anchors and the inhibition of cytoplasmic influx, (3) cationic amino acids (such as arginine) to augmente electrostatic interaction with cellular membranes, and (4) cancer recognition moiety to facilitate membrane–mediated recognition of target cancer cells.

**Fig. 8:**
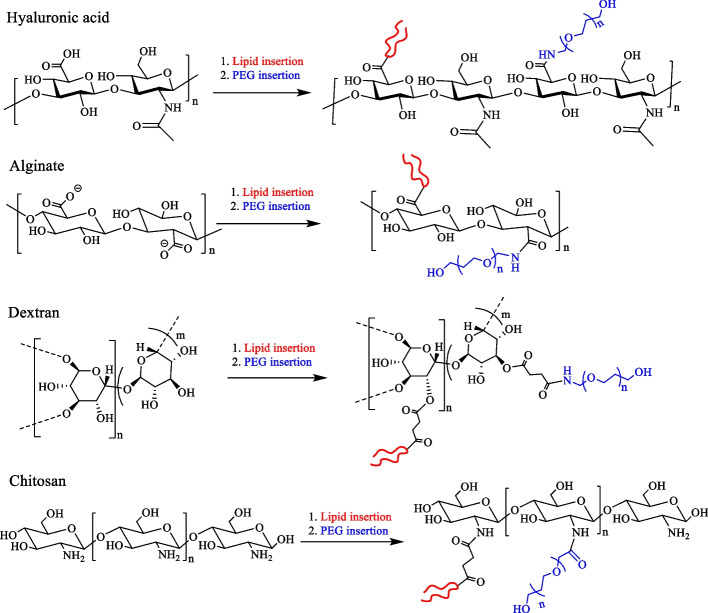
Schemes representing the modification strategies of natural biopolymers for designing two–tail lipid-based cell–coting platforms

**Fig. 9:**
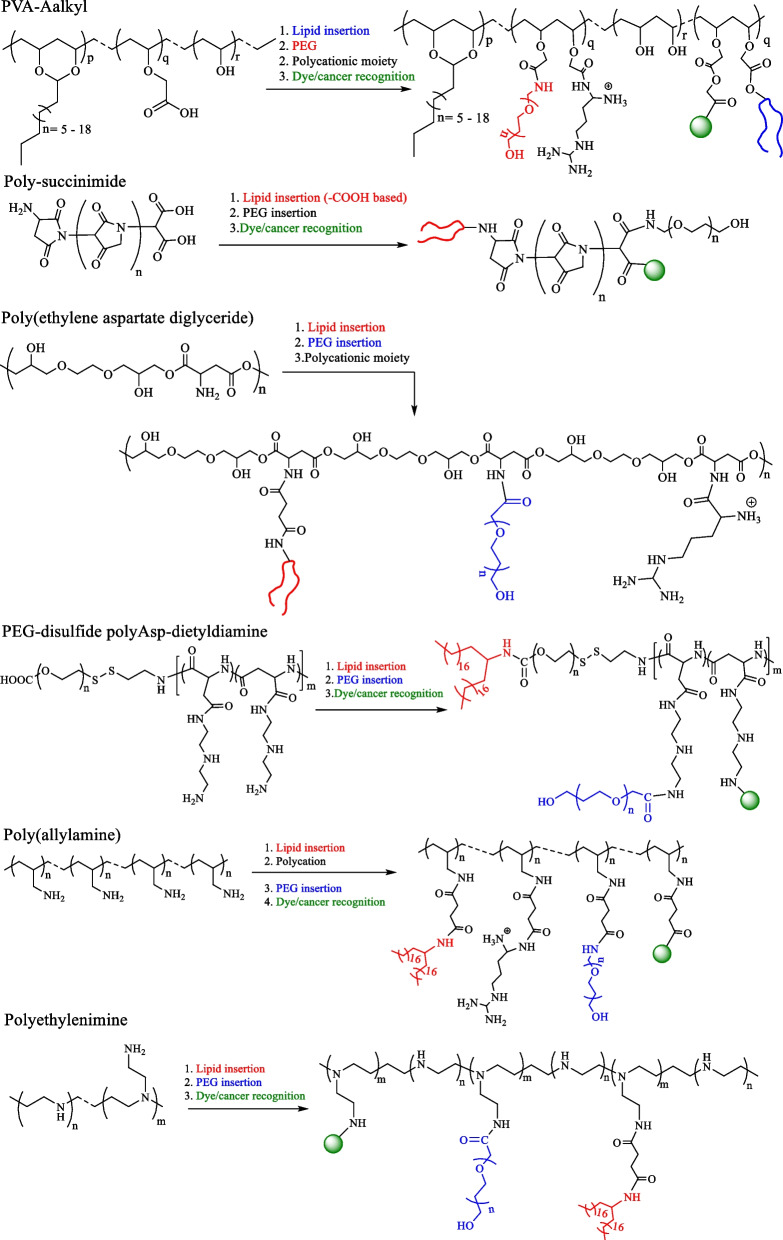
Schemes representing the modification strategies of synthetic polymers for designing cell–coting platforms

Figures 8 and 9 represent possible design approaches for natural and synthetic polymeric anchors for NK cell–coating materials. The incorporation of such modular moieties could both offer sufficient localization onto NK cell membranes, and facilitate target recognition, and eventually enhance anticancer efficacies of surface–modified NK cells to treat a series of cancers. The addition of PEG also showed higher immune response and the prolonged persistence of targeted moieties [161, 162].

Natural polysaccharides boost the immune system and help to combat several ailments [77]. Polysaccharides, which are closely related to immune regulation, mainly increase the activity of immune cells, promote the secretion of cytokinin, and enhance immune functions. Polysaccharides also enhanced the activity of NK cells and macrophages to increase phagocytosis toward foreign particles [163]. In addition, the overexpression of MMPs in tumor tissues remodulates the ECM to promote metastasis. The use of bioactive polysaccharides could also inhibit metastasis by the suppression angiogenesis and inhibit the activity of MMPs [164]. Polysaccharides, like hyaluronic acid and chondroitin sulfate, can easily avoid the ATP binding and could directly target CD44 overexpressing cancer cells [165, 166]. Polysaccharides are widely used for anticancer drug delivery applications to bypass the side effects of many drugs. Polysaccharides may also stimulate the intrinsic anticancer immune system themselves. In this regard, many polysaccharides, such as chitosan, hyaluronic acid, dextran, inulin, sodium alginate, and other polysaccharides have been explored as potential drug delivery systems, and as well for immune adjuvants for cancer treatment via the promoting release of interferon–γ and granzymes [167–170]. The polysaccharides also regulate the immune mechanism via different pathways, including NF–κB, TLR4, and notch pathways. These pathways have major influence on NK cells, dendritic cells, and macrophages to improve the immune microenvironment [171]. Polysaccharides are highly safe for human applications; however their rapid elimination, short half-life, and lack of specific binding hinder their pharmacological activities. Some researchers maintain that the proper modifications of polysaccharides have more immunomodulatory activity than those without functional moieties [167].

Therefore, Fig. 8 demonstrates the developments of next-generation polysaccharides-based biomaterials for cell–coating applications. Polysaccharide backbone could be a framework for multiple chemical conjugation along with inducing interaction with membranes of target cancer cells.

For example, functionalized chitosan-based materials have been developed for plasma cell membrane coating [172]. With the aid of cholesterol as hydrophobic anchoring moiety, the surface integration of the resultant materials maintained integrity up to 6 h without cellular internalization. Another example of chitosan–cholesterol–biotin was also applied for plasma cell membrane coating, and showed stable surface anchoring up to 8 h [173]. Therefore, the incorporation of natural polymer backbone with hydrophobic lipid anchor could be an innovative strategy for cell surface modification and labeling to prolong cell membrane behavior. Similarly, synthetic block–copolymers have also been used for cell–coating materials (Fig. 9**)**, for the purpose of enhancing cellular activities including migration of stem to injured tissues, hiding transplant cells from immune response, and promoting natural differentiation in stem cells [174–176]. While block–copolymer incorporation can occur without modification of the cell membrane, such modification can disturb the functions, compared to the therapeutic efficacy of cells. Therefore, the insertion of two–tailed hydrophobic lipid segments shows rapid cell membrane anchoring.

Block–copolymers modified with functional moieties can easily impact cell membrane and these materials can be utilized to improve cell-based therapies. In recent years, modified block-co–polymers have been applied for cell membrane modification. For example, poly(oxanorbornene)-based block copolymer conjugated with Brij moiety was anchored on the living Jurkat cell membrane. The synthesized Brij-based copolymer was introduced on the periphery of cell membrane, without showing any toxic effects up to 24 h of incubation. The polymer concentration below the critical micelle concentration (5 µM) was used to anchor at the 3T3 fibroblast cell membrane to facilitate homogenous surface coating without internalization. The flow cytometry results suggested the surface coating of the cell membrane without altering the cell membrane and cellular internalization. After successfully coating the cell membrane, the polymers did not show cytotoxicity after 24 h of incubation. Additionally, photosensitizer molecule conjugated into this copolymer was used to generate localized ^1^O_2_ near the cell surface for trigger cell death without harming healthy cells [177]. Living cell microencapsulation can provide alternative methods to develop new therapeutic effects. Unfortunately, due to the large size of microencapsulated cells, they have mass transportation issues, and clinically are not suitable for veins [178]. The transplantation of microencapsulated islet cells shows limited vascular supply, which generates a hypoxic cell condition [179]. Therefore, to avoid this issue and minimize the capsule size of encapsulated islet cells, Willson et al., utilized a layer–by–layer approach for intraportal islet transplantation. The poly (L-Lysine)-g-poly(ethylene glycol) conjugated with biotin (PBB) was synthesized, and used for the islet cells surface modification through a layer–by–layer cell–coating method using streptavidin, after the successful layer-by-layer surface coating of islets using a PBB copolymer that facilitates cell surface growth without toxicity and loss of cell functions. Therefore, the nanothin coating of islets cells provides a novel approach, and reveals an informative relationship between cationic charge density, cell membrane attachment and biocompatibility for targeted cell delivery applications [180].

## Anchored NK cell mediated biomedical applications

Surface-functionalized NK cells have highly advantageous properties towards targeting malignant tumors by generating facilitated immune synapse interaction and cell–cell binding [181]. Subsequently, modified NK cells showed the enhanced therapeutic potentials of upregulated target recognition and cancer killing efficacy against various tumors. Therefore, the effective surface presentation of functional moieties (such as glycan, nanoparticles, aptamers, and antibody) onto NK cell membranes has been developed, as described in this section.

### NK cells surface engineering using glycans

The glycoengineering of NK cells can provide precise targetability and desirable cell recognition. Glyco analogs show high specific affinity with Siglecs target cell membrane. For example, sialic acid analogs, like C9 of Neu5Ac, -N-biphenylcarboxamide-Neu5Ac and 9-N-m-phenoxybenzamide-Neu5Ac showed specificity and high affinity with CD22 [182]. Cancer cell overexpressed inhibitory ligands could avoid immune cell detection and following attack. For example, sialylation on cancer cells disturbs cell–cell interaction between NK cells and tumor cells.

The glycol-based molecules have been added by both direct and indirect conjugation at the cell membrane surface [183–185]. These processes can also be achieved by both covalent and non-covalent processing. Linker molecules are often required to conjugate different functional groups including maleimide, azide, hydrazide, and alkynes. However, the direct glycan conjugation strategy onto cellular surfaces still has a few drawbacks, such as low binding efficiency and transient nature [186].

**Fig. 10:**
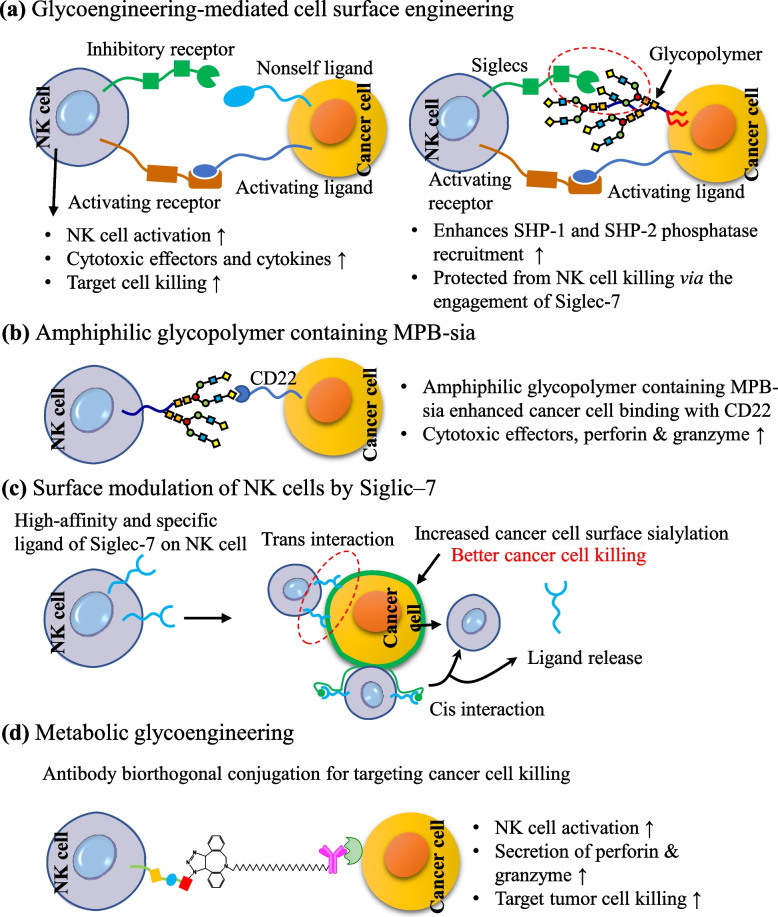
Physical attributes of different glycans at NK cell surfaces affecting the immunological functions to augmented cancer cell killing. **a** Glycoengineering approach for cancer cell membrane modification to prevent killing from NK cell, (**b**) glycomodulation of NK cells via CD22 specific ligands, (**c**) surface modulation of NK cells by Siglic–7, and (**d**) metalbolic glycoengineering of NK cells for antibody-mediated targeting cancer cell killing

To enhance glycan-mediated cell–cell interaction, a few strategies have also been suggested whereby a glycocalyx engineering approach could be applied to elucidate the roles of specific sialosides in facilitating Siglec–based immunoevasion. The tumor cells engineered with sialylated glycopolymers are prevented from NK cell killing due to strong Siglec binding (Fig. 10a) [86].

CD22 highly expressed various type cancer cells and it can be selectively targeted by Sigleic–2 glycan. To achieve tumor-specific CD22 targeting, highly specific Neu5Ac analogue ligands were conjugated with NK–92MI cells through chemoenzymatic generation to enhance the therapeutic efficacy of the cell for better cancer treatment. The two unnatural Neu5Ac analogues, CMP–^BPC^Neu5Ac and CMP–^MPB^Neu5Ac conjugated on NK–92MI cells show 20–50 fold higher binding affinity with the CD22 ligand in comparison to CMP-Neu5Ac to install the natural ligands [187]. The modified sialoside analogues containing heterocyclic ring moieties also show high affinity and selectivity towards siglecs. For example, the addition of triazole, benzamide, acetylene, azides, and dimethyl benzamide substituent in sialosides through click chemistry improved the affinity and selectivity toward CD22 and CD33 overexpressed cells [188].

Glycoengineering with 9-*O* modified sialic acid of NK cells, can significantly improve the selective binding with Siglec–2 B-cell-restricted antigen CD22^+^ cells. The addition of MPB-sia group through the sialic acid biosynthetic pathway greatly enhanced the binding affinity with CD22^+^ cells. The glycoengineered NK cells represent CD22–dependent cytotoxicity against CD22^+^ primary lymphoma cells (Fig. 10b) [189].

Chemoenzymatic glycans (ST6Gal1) surface editing of NK cells shows high affinity and specificity toward the modulation of Siglec–7 signaling. The ST6Gal1-mediated surface glycan was added to the NK cell surface to generate cis-type interaction toward target cell. The higher glycan ligands on the NK cell surface enhanced Siglic–7 phosphorylation and SHP–1 release, which suppresses the NK–induced target tumor killing while lower-level help to release Siglec–7 which helps to restore NK cell killing efficacy. Hence, low level of high–affinity ligands in *cis* manner destroys Siglec–7 clusters which release Siglec–7 through secretion pathways and triggered immune killing efficacy (Fig. 10c) [190].

The ex vivo glycoengineering with IL–21on the NK cell surface induced effective antitumor targeting and infiltration, and enhanced autoimmune response and potential therapeutic response. IL–21 is an imperative regulatory marker that can efficiently activate and start transcription factors to modify autoimmune reactions. However, the attachment of IL–21 to NK cells by tradition nal methods has a week effect on NK cells. Therefore, tumor–specific release IL–21 nanoparticles were designed to enhance the activation, expansion, and safety performance of NK cells. Bio-orthogonal chemical groups (N_3_ and BCN) were separately incorporated on the surface of NK cells. After that, cytokinin IL–21-based redox responsive nanoparticles were conjugated on –N_3_-containing NK cell surface. Finally, bio-orthogonal live-cell nanoparticles augmented the interaction between NK and target cell and the in situ controlled release of IL–21 from nanoparticles, promoting the more effective and therapeutic efficacy of NK cells [191].

Similarly, metabolically glycoengineered NK cells conjugated with antibodies through biorthogonal reaction were used to efficiently enhance targeted cancer killing efficacy. The NK cells were glycoengineered with 9-azido N-acetyl neuraminic acid methyl ester (N_3_–SA), and generated the surface glycan with a free azide group. Elsewhere, a monoclonal antibody (mAB) was separately conjugated with DBCO–PEG4–NHS ester to generate mAB–DBCO (Fig. 10d). The synthesized mAB–DBCO was further connected with N_3_–NK cells through biorthogonal reaction. The glycoengineered antibody-based NK cells demonstrated better binding efficacy with EGFR positive KRAS mutant SW480 human colorectal cancer cells as compared to native antibodies and uncoated NK cells [192].

### Nanoparticle-mediated NK cells

The physical specification of nanomaterials can modulate the NK cell immune response for cancer treatment. The nanomaterials design strategies and specifications, like structure, surface charge, valency, and hydrophilic/hydrophilic nature, can be designed to induced potential immune response between NK cell and cancer cells [193]. Nanoparticles play an important role in the activation of NK cells, and also act as a good immunomodulator to increase immunotherapeutic efficacy. Nanoparticles shown different pharmacodynamic and pharmacokinetic properties and lower dose of nanoparticles somehow modulates the immune system with minimum side-effects [194].

**Fig. 11:**
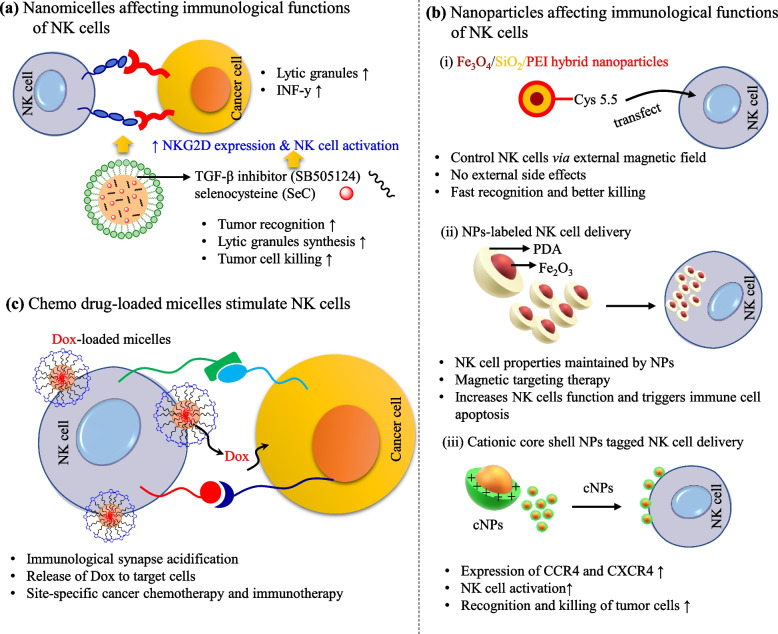
The nanomaterials affecting the immunological functions of NK cells. **a** A nanomicelles system activating the NK cells and augmenting NK cell functions for cancer treatment, (**b**) physical attributes of different nanoparticles altering the immunological functions of NK cells, and (**c**) Acid-responsive chemo drug-based micelles immune synapse generated by the release of dox in acidic environment

The tumor killing vitality of NK cell was influenced by the tumor–secreted regulators. For example, tumor secreted TGF–β is a negative regulator for IFN–γ production by NK cells. TGF–β also downregulates the NK cell activation receptors, like NKp30, NKp46, and NKG2D, which reduced the toxicity of NK cells toward tumor cells. Park et al. reported a strategy to modulate TGF–β signaling using nanoscale liposomal gel, which showed tumor killing efficacy through NK cell activation. Nanoscale liposomal gel potentially inhibits TGF–β signaling, and successfully delivered the IL–12 into the tumor site. The results of this study demonstrated the delivery of hydrophobic and hydrophilic immunomodulators to enhance cancer killing efficacy against melanoma and breast cancer cells [195]. Similarly, Liu et al. designed a nanoemulsion system for the co-delivery of TGF–β inhibitor and selenocysteine (SeC) to enhance NK cell tumor-killing efficacy against triple-negative breast cancer cells. The designed nano emulsion system effectively downregulates TGF–β/TGF–β RI/Smad2/3 signaling and enhance immune response through NKG_2_DL expression on the cancer cell and NKG_2_D expression on the NK cell surface (Fig. 11a). The site-specific release of TGF–β inhibitor and SeC significantly increased the antitumor efficacy against breast cancer [196].

Recent nanotechnology technologies further enable the development of a variety of nanoparticle systems to achieve better therapeutic efficacy of NK cells [197]. Therefore, the surface modulation of NK cells using nanoparticle system is also useful for NK cell homing and infiltration to achieve efficacy of NK cell cancer immunotherapy. Recently, various nanoparticle-based strategies showed promising NK cell tumor infiltration, where NK cells stimulate the production of pro-inflammatory cytokines, and induced cytotoxicity against tumor cells [198, 199]. In addition, the nanoparticles attached to the NK cell surface can also control the moment of NK cell after local delivery using an external magnetic field. For example, Jang et al. developed Cys5.5–conjugated Fe_2_O_3_/SiO_2_ nanoparticles for modification of the surface of NK cells [200]. Cys5.5 Fe_2_O_3_/SiO_2_ was attached to NK cells, and modified Cys5.5 Fe_2_O_3_/SiO_2_@NK cells were used for cancer recognition and tumor targeting (Fig. 11b). Magnetic field induced increase in the relative ratio of NK cells infiltrated into tumor tissue by 17 times, even at the minimum concentration of nanoparticles (20 μg Fe/mL), which is relatively lower than conventional magnetic resonance monitoring agents. Therefore, the results were explored to apply a cell-based therapy with MRI imaging for tumor treatment without surgery. On other hand, Wu and coauthors synthesized biocompatible polydopamine-coated Fe_3_O_4_ nanoparticles (Fe_3_O_4_@PDA), which were encapsulated by NK cells [201]. The synthesized Fe_3_O_4_@PDA nanoparticles were successfully internalized into NK cells without affecting the intrinsic properties of NK cells. The presence of markers at the NK cell surface indicates the biocompatibility of nanoparticles, and hence induced the maturation of NK cells. The Fe_3_O_4_@PDA–loaded NK cells significantly induced apoptosis against A549 cells and inhibited tumor growth as compared to unmodified NK cells. Fe_3_O_4_@PDA loaded NK cells also pointedly reduced the Ki67 + tumor cells with an increasing number of apoptotic cells (Fig. 11b). In another study, the NK cell surface was engineered using iron oxide nanoparticles (IONPs) to localize delivery to the desired targeted site of action under a magnetic field [202]. IONPs were immobilized on the NK cell surface using a robust bioconjugation technique by attaching sulfo–NHS biotin to the surface amine, and attached streptavidin–coated IONPs. The activity of NK cells, including phenotype and functions, was maintained over time. In addition, the bio-hybrid therapeutic approach was successfully homed with magnetic response to improve antitumor efficacy in contrast to naked NK cells.

Recently, nanoparticle-mediated photothermal strategies have attracted great attention for cancer immunotherapy. Photothermal therapy is oxygen–independent, hence it can be used efficiently to kill solid tumors by overcoming tumor hypoxic conditions. Nanoparticles-based photothermal strategies generated heat by inducing infrared irradiation, and were able to target tumor tissues, and have desirable tumor–killing efficacy [203]. The nanoparticle photothermal agents promote the development and infiltration of cytotoxic CD8^+^ T cells through pro-inflammatory cytokines release. Therefore, the nanoparticulated photothermal agents provide an active immune response in addition to reversing the TME's immunologically "cool" state [204]. Combined photo–immunotherapy offers a promising therapeutic approach. For example, Zhang et al. produced 2D manganese-based coordination nanosheets (CONASHs), and then the attachment of polyether imide and DNAzyme; the DNAzyme@Mn–CONASHs were anchored on the surface of the NK cell [205]. Further, the TLS11a aptamer was conjugated with DNAzyme@Mn–CONASHs@NK through a glycan biosynthesis approach. The synthesized platform was homogenously anchored on the surface of NK cells, which was confirmed by CLSM images labeled with Cys3–TLS11a aptamer–DBCO. The stability of the TLS11a aptamer in NK cells was controlled for up to 24 h. In addition, the morphology of HepG2 cells that were treated without TLS11a aptamer NK cells did not change, but when treated with NK cells decorated with TLS11a aptamer, were significantly changed. After in vivo* injection into* mice, the DNAzyme@Mn–CONASHs based on NK cells showed better tumor inhibition compared to the case for DNAzyme@Mn–CONASHs alone.

Cationic biomaterials are transfection agents, that primarily activate and trigger immune cells like macrophages, dendritic, or other immune cells (Fig. 11b). Cationic biomaterials also demonstrate adaptive immune responses by promoting immunity with stimulation to produce porin-inflammatory cytokine by immune cells. For example, PEI, a cationic polymer shows a tendency to activate macrophages through TLR–4 interaction. Elsewhere, the intratumoral injection of PEI increases the expression of IL–12, and decreases the expression of IL–10 [206, 207]. Kwang-Soo Kim and coauthors synthesized PEI–immobilized PDA–coated Zn/Fe magnetic nanoparticles (41 nm) for NK–92MI cell activation. The synthesized magnetic nanoparticles did not show toxicity against NK cells, and successfully enhanced cytolytic activity against triple-negative breast cancer cell [208]. In other research, Im et al. designed a pH-responsive micelle system for the site–specific delivery of chemo drug (DOX) through immunotherapy (Fig. 11c). When the micelle-NK cells were cultured with cancer cells, the pH between NK and cancer cells decreased, due to releasing lytic granules from NK cells. Therefore, the interaction between NK, and cancer cells generates acidic conditions that triggered the site–specific release of chemo drug [209].

### Aptamer-immobilized NK cells

Aptamers are the 3D structure of DNA/RNA oligonucleotides, that can specifically bind to selective proteins. Currently, aptamers are widely well-accepted ideal candidates for the targeted delivery, diagnosis, and immunomodulatory agents of cancer. A great benefit of aptamers is their facile chemical modification and molecular engineering. The chemical modification used aptamers prior to enhancing the stability against serum, and enhanced targeting specific binding ability [210]. In cancer immunotherapy, aptamers allow for simultaneous cancer cell recognition, and induced immune synapse. Aptamers show high affinity with various co-inhibitory immune checkpoints including CTLA–4, PD–L1, PD–1, TIM–3, and LAG–3. Aptamers also activate the immune cells through INF–γ release to prevent tumor growth [211–213].

**Fig. 12:**
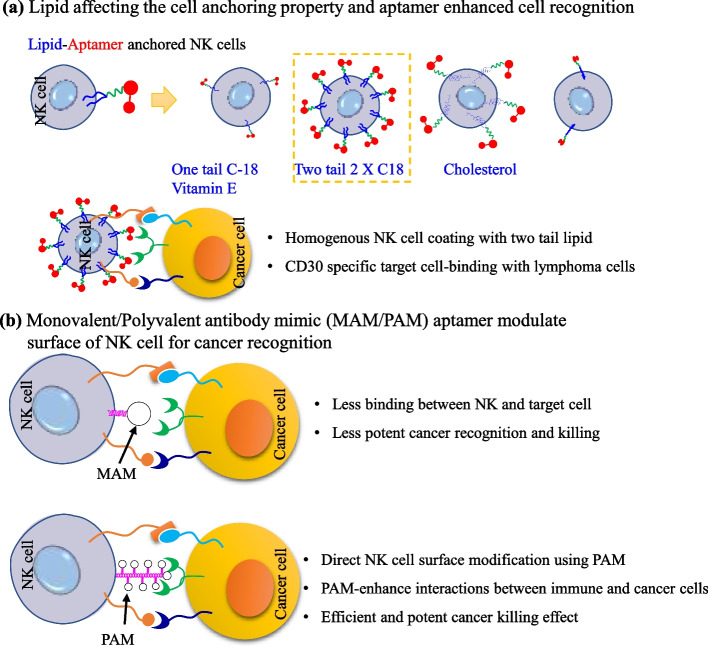
Aptamer-based NK cell modifications (**a**) Lipid-based different aptamers NK cell anchoring and CD30 specific targeted cell binding, and (**b**) direct NK cell functionalization using aptamers for specific cancer cell recognition

To develop aptamers functionalized NK cells for targeted cancer immunotherapy, Yang et al., synthesized CD-30 specific aptamer-based different lipid anchors including single and double chains. As mentioned in Fig. 12a, different lipid moieties, like an 18–C single chain, 18–C × 2 two-tailed lipid, cholesterol, and vitamin-E were used for the CD–30 specific aptamer conjugation. After 30 min incubation of lipid–aptamer with NK cells, aptamer–18–C × 2 two-tailed lipids showed good and homogeneous surface coating without the intact cell morphology of NK cells. Owing to the absence of intracellular signaling domains, the 18–C × 2–NK cells showed potential cell–specific binding without affecting the activity of NK cells, and enhanced targeted cell killing against lymphoma cells for improved immunotherapy [214].

As NK cell-based therapeutic medicine circulates in the body with certain shear conditions before reaching the targeted site of action, hence the body shear flow environment can significantly influence cell–cell interactions [215, 216]. Therefore, NK cells coated with multifunctional biomaterials can control the cell–cell interaction in shear stress conditions. Multivalent aptamers also have an advantage in targeted immunotherapy under body shear conditions. The benefit of the multivalent aptamer is the ability to direct focus on immune signals to the cell group of interest. To develop cell–cell interaction, one partition of aptamer can bind to tumor cell surface antigen or in TME, and another partition can bind with the specific receptors of immune cells. Therefore, to prove the concept of multivalent aptamer mimicking cell–cell interaction under shear stress, Shi and coworkers synthesized in situ aptamer–based polyvalent antibody mimic (PAM) and monovalent antibody mimic (MAM) for cancer cell targeting and enhance cell–cell binding. The synthesis of polyvalent aptamers involved three major steps: (1) DNA initiator on cell surface, (2) the formation of supramolecular DNA scaffolds on the cell surface, and (3) the direct connection of DNA scaffolds with aptamers (Fig. 12b). The PAM@NK cells are more capable than MAM@NK cells in recognition and binding to K562 cancer cells under shear stress conditions [216].

### Antibody-dependent NK cell-mediated cytotoxicity

The concept of antibody for targeted therapy (magic bullet) was first advanced by Paul Ehrlich [217] who suggested the specific detection and suppression of malignant tissues. Five sequence-based antibodies like IgG, IgM, IgD, IgE, and IgA are found in two different subunits: (1) constant fragment and (2) fragment of antigen binding. The constant fragment antibodies are linked with immune effector functions and show binding to IgG receptors (FcyRs) and neonatal FcR (FcRn) [218]. These antibodies can sufficiently interact with the overexpressed surface receptors of tumors, accumulate into tumor tissues, and significantly inhibit tumor growth. The interaction between antibody and tumor receptors allows immune effector functionalities like Fc receptors overexpressed by immune cells [219].

**Fig. 13:**
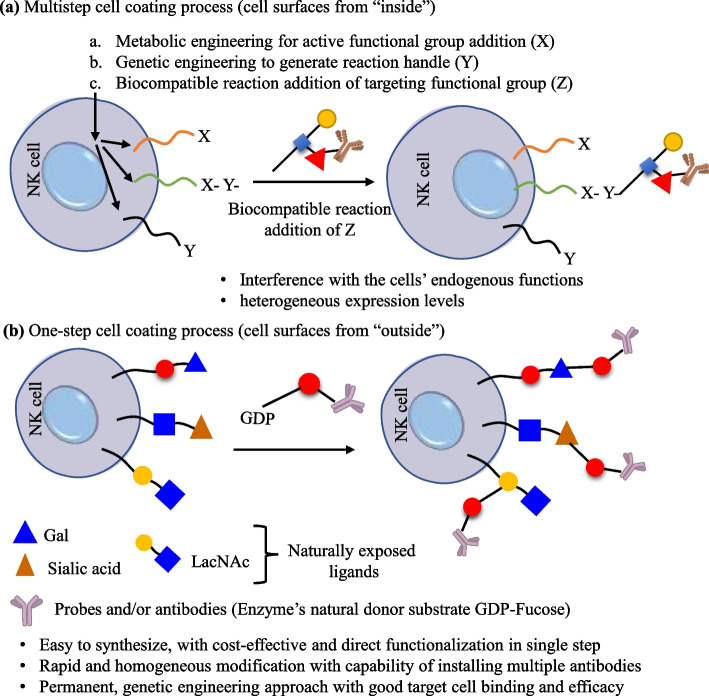
**a** Scheme representing the multistep cell–coating process and affecting functions of NK cells and (**b**) one-step NK cell–coating process augmented the NK cell functionalities and targeted cancer cell binding

**Fig. 14:**
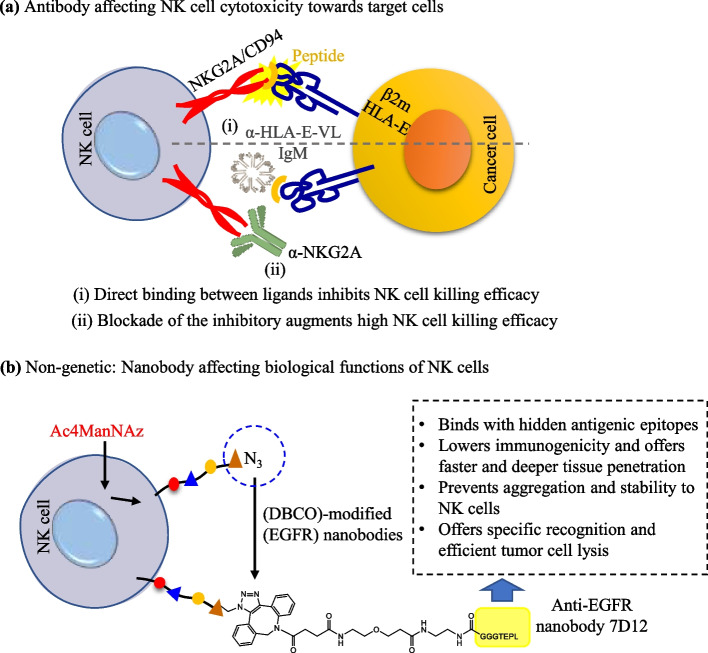
**a** Antbody affecting the binding between NK and cancer cell, and (**b**) non-genetic NK cell surface modification approach for effective cancer targeting

The unconjugated antibodies have lower immune effector function efficacy, hence modification via chemical or genetic agents may increase the targeted immunotherapy. Mainly, the capacity of antibodies to generate adaptive immune effectors such as NK and T cells can induce anticancer activity. In terms of NK cells, the overexpression of phosphatase motifs (SHP-1/2 or SHIP) also inhibits the immune system [220]. Therefore, systematic antibody modification or conjugation may expand the ability to stimulate immune effector functionalities [221–223]. In past years, the antibody–dependent engagement of immune mechanisms has been used for cancer cell killing. However, during the multistep cell–coating process, the addition of antibodies through genetic engineering has some technical complications and safety issues. The major limitation is interference with cell endogenous functions during surface modification (Fig. 13a). Therefore, antibody-based surface engineering at the cell membrane using chemo-bio tools in single-step has emerged as a complementary and useful technique (Fig. 13b). In the Jie Li and coworkers study, NK cell surface was engineered using GDP Fucose-conjugated human IgG (GF–hIgG) antibody with Herceptin to target HER2 + breast cancer [224]. The half-life of Herceptin was maintained up to 20 h, and it selectively binds with BT474‒, a HER2 + breast cancer cells, via cluster formation. These surface–modified NK cells with Herceptin were shown to produce more than 7–times lysis of BT474 cells, as compared to the unmodified NK cells. The killing effects of Herceptin–NK cells were also confirmed in other HER2 + cells that showed potential cell-mediated cytotoxicity with effective targetability in ex vivo and in vivo death. In another study, Dapeng Li and coauthors had 3H4 and CA147 IgM antibodies, and utilized them for NK cell surface engineering [225]. The modified NK cells efficiently bind with HLA–E–VL9 and inhibit NKG2A/CD94 which suggested the 3H4 IgM enhances NKG2A + NK cell death. Finally, this study suggested that mouse 3H4 IgM and human CA147 antibodies potentially work as an NK checkpoint inhibitor, and target HLA–E–VL9 (Fig. 14a).

Moreover, Gong and coworkers synthesized anti–EGFR nanobody 7D12 for cancer cell targeting as an alternative genetic approach method. NK92MI cells were metabolized to generate azo-modified sialic acid, and it was then further conjugated with aza–DEBCO-modified anti-EGFR nanobodies by bio–orthogonal click chemistry method [226]. After that, 7D12NK cells were applied in the different EGFR^+^ tumor cell lines, including MDA-MB-468, A549, A431, LoVo, and RKO cells. The developed therapeutic approach 7D12NK cells demonstrated active targeting toward EGFR overexpressed cancer cells. This new type of biotherapeutic approach is capable of penetrating solid tumor tissues, with low immunogenicity and improved therapeutic efficacy of the NK cells in both ex vivo and in vivo treatments **(**Fig. 14b**)**.

### Conclusion and future perspectives

The development of novel biomaterials is being pursued to satisfy the urgent requirements of cell–based therapies with their unique performance. In recent years different cell-coating strategies have been used for the development of cell-based therapies including CAR–based therapy. The cell–coating techniques successfully tuned the surface properties of cells like cell tracking, imaging, and cell–cell interactions. Controlling cell surface engineering can be achieved by modifying a cell membrane with an active polymer. In general, three major types of polymers have been used in cell surface engineering, namely: (1) ionic polymers for layer-by-layer deposition, (2) reactive functional polymers for direct surface functionalization, and (3) amphiphilic polymers for hydrophobic insertion. In the following context, the utilization of these materials and their perspective application to augmented cell functionalities are briefly discussed. This review has introduced different types of polymeric biomaterials for controlling NK cell behavior, a detailed discussion of surface functionalized NK cells with their potential anticancer applications, and the state-of-the-art in the design of some new biomaterials to alleviate NK cell surface behavior for cancer immunotherapy. Some most common important cancer recognition moieties including folic acid, lactobionic acid, and phenylboronic acid, and their potential applications for cell surface utilizations are also discussed. Elsewhere, most of the currently developed cell-based methodologies and modified NK cells with biomaterials and/or other active templates are introduced. To resolve the inevitable cell–cell interactions by the conventionally available methods, new strategies have also been proposed for large-scale development of biomaterials for cell–surface modifications.

Advanced biomaterials synthesis techniques and their post-functionalization allow for the rational design and effectiveness of polymers toward new cell-based therapies. For example, biomaterials functionalized with lipid, PEGs, and cancer recognition moieties can represent great potential for immune cell surface engineering and immunotherapy applications. In particular, hydrophobic insertion and cancer recognition moiety can enrich the surface functionalities of NK cells for cancer treatment. Along with advancements in biomaterials functionalization, various architectures can precisely enhance the NK cell surface functionalities. Controlled conjugation and specific topology of polymeric biomaterial can be anchored on the NK cell surface with tunable and unique surface properties towards cancer treatment applications. However, the disadvantages of layer-by-layer and direct conjugation of materials with cell membranes cause many side effects. Elsewhere, the CAR–NK cell-based therapy is also a cost-effective technique. Focusing on this issue, the functionalization of different biomaterials has been proposed to develop surface-engineered immune cells for cancer immunotherapy applications. The development of a new cell-based therapy system has brought good hope for cancer patients. Cell surface engineering methodologies have significantly improved the therapeutic abilities of NK cells through different sources of functional motifs. Cell surface modification improves the protection of cells from harsh tumor microenvironments, inducing cell–cell communication, stability, and effective targeting ability without interfering with the intrinsic properties of cells. Therefore, the surface engineering of NK cells using multifunctional biomaterials is a new predictable concept for the development of novel cell-based therapeutics systems for solid tumor treatment.

## Data Availability

Not applicable.

## Abbreviations

CAR: Chimeric antigen receptors
NK: Natural killer
TRAIL: Tumor necrosis related apoptosis induced ligand
FasL: Fas ligand
EPR: Enhanced permeation and retention
FA: Folic acid
ASGPR: Asialoglycoprotein receptors
LBA: Lactobionic acid
PD-1: Programmed cell death protein 1
PD-L1: Programmed death-ligand 1
ITIM: Immunoreceptor tyrosine based inhibitory motif
PBA: Phenylboronic acid
PEG: Polyethylene glycol
PVA: Polyvinyl alcohol
PEI: Polyethylene amine
DMPE: 1,2-Dimyristoyl-sn-glycero-3-phosphoethanolamine
DPPE: 1,2-Dipalmitoyl-sn-glycero-3-phosphoethanolamine
DSPE: 1,2-Distearoyl-sn-glycero-3-phosphoethanolamine
ECM: Extracellular matrix
MMP: Matrix metalloproteinases
DBCO: Dibenzocyclooctyne
IFN-γ: Interferon gamma
PAM: Polyvalent antibody mimic
MAM: Monovalent antibody mimic
